# The impact of the COVID-19 pandemic on the well-being of autistic and non-autistic adults in Eastern Germany

**DOI:** 10.1186/s12888-025-07430-x

**Published:** 2025-10-02

**Authors:** Josefine Rothe, Tobias Thiel, Veit Roessner, Melanie Ring

**Affiliations:** 1 Department of Child and Adolescent Psychiatry, Medical Faculty & German Center for Child and Adolescent Health (DZKJ), partner site Leipzig/Dresden, Technische Universität Dresden, Dresden, Germany; 2 Department of Child and Adolescent Psychiatry, Medical Faculty, Technische Universität Dresden, Dresden, Germany

**Keywords:** Mental health, Autism, Adults, COVID-19

## Abstract

**Background:**

Autistic individuals often experience significant difficulties adapting to even slight changes in their routines. The COVID-19 pandemic created an uncertain situation marked by frequent changes in daily life, though some pressures of everyday life were reduced during this time. The few existing studies on mental health in autistic individuals during the COVID-19 pandemic have identified a link between pandemic-related distress and increases in symptoms of anxiety and depression. The present study aimed to compare the impact of pandemic-related restrictions on daily life and psychopathological symptoms between groups of autistic and non-autistic adults and to determine whether these variables are interrelated. Furthermore, the study examined potential predictors of psychopathological symptoms during the pandemic.

**Methods:**

A sample of 86 East German autistic adults aged 18–67 years (21 female, *M*_age_ = 33.49 years, *SD*_age_ = 13.32) and 87 non-autistic adults aged 18–70 years (21 female, *M*_age_ = 34.37 years, *SD*_age_ = 14.18) completed self-report questionnaires addressing autism-like traits, the impact of pandemic-related restrictions on daily life, psychopathological symptoms, sensory sensitivity and Intolerance of Uncertainty (IoU). The 7-day incidence rate, pandemic duration, and the scope of social restrictions at the time of the survey were considered individually for each participant.

**Results:**

Regarding pandemic-related restrictions, the reduction in social contacts and restrictions on freedom of travel were most relevant for both groups while non-autistic adults reported that they were affected more severely compared to autistic adults. Retrospective self-ratings of changes in overall physical and mental health did not differ between the two groups. Consistent with pre-pandemic evidence, autistic adults reported higher sensory sensitivity and greater IoU compared to non-autistic adults. However, sensory exposure due to face coverings affected both groups similarly. Sensory sensitivities and IoU were the most relevant predictors of psychopathological symptoms in both groups, although changes in overall physical health also emerged as a predictor for autistic adults.

**Conclusions:**

The relation between pandemic-related restrictions and the predictors of psychopathological symptoms differed somewhat between groups. Although it is known that autistic individuals show higher levels of sensory sensitivity and IoU in general, autistic adults appeared to be less affected by certain pandemic-related restrictions than anticipated.

**Supplementary Information:**

The online version contains supplementary material available at 10.1186/s12888-025-07430-x.

## Background

Next to the well-known core features [1], autism is often accompanied by additional medical and psychological challenges, which occur at rates 3–4 times higher than in non-autistic individuals [2]. Concerning co-occurring mental disorders, a recent systematic review revealed particularly high lifetime prevalence rates for anxiety (42%) and depression (37%) for autistic individuals [3]. Social phobia and obsessive-compulsive disorders are among the most common anxiety disorders in autism [3].

Several factors have been identified as predictors of symptoms of anxiety and depression in autism, including Intolerance of Uncertainty (IoU) predicting symptoms of anxiety and depression as well as sensory sensitivity predicting symptoms of anxiety [4–10]. Sensory sensitivity is now recognized as a core feature of autism within the domain of restricted and repetitive behaviours [1] and encompasses over- as well as under-sensitivity to certain stimuli. IoU refers to the tendency to perceive uncertain situations as aversive, leading to efforts to avoid such contexts. IoU has been found repeatedly to be higher in autistic than non-autistic individuals [4, 11]. Our recent findings on a model predicting anxiety symptoms in autistic and non-autistic adults demonstrated significant differences in the predictive patterns of IoU and sensory sensitivity between both groups and, therefore, emphasised the relevance of both factors for understanding the mechanisms of anxiety in autism [5]. However, the question remains to what extent these two factors are also relevant for other co-occurring mental disorders (e.g. depression, obsessions and interpersonal sensitivity) and whether the prediction patterns also differ between autistic and non-autistic adults for such co-occurring mental disorders. In this context, previous studies showed that IoU was a rather global predictor relevant for a broader range of individuals and psychopathologies [4–10, 12]. An example of an uncertain situation that affected society as a whole was the COVID-19 pandemic, characterized by almost daily fluctuations in infection rates and constantly changing regulations. Since autistic individuals often perceive even minor changes as unsettling and struggle with slight routine disruptions, such an uncertain situation was likely far more distressing for them than for non-autistic individuals. At the same time, it is conceivable that some of the pressures of daily life autistic individuals struggle with may have been alleviated at the time of the COVID-19 pandemic – for instance, the pressure of interactions with other people which was reduced partially during lockdowns by the social restrictions. While most studies at that time focussed on the experiences of autistic children and their caregivers, only few investigated the situation of autistic adults finding mixed results. A survey of autistic adults and their caregivers found that participants reported a reduction in stress and a decrease in various areas of psychopathology among autistic adults [13]. Most other studies found an increase in psychopathology in autism such as the survey of Oomen and colleagues [14], where autistic as well as non-autistic adults reported an increase in symptoms of anxiety and depression following the onset of the COVID-19 pandemic, with a more pronounced increase in the autistic population. Autistic adults expressed concerns about the loss of their routines but also relief due to a reduction in sensory input and social demands. Similarly, Nistico and colleagues [15] reported an increase in symptoms of anxiety, depression, post-traumatic stress disorder and stress among Italian autistic compared to non-autistic adults during the first lockdown. These symptoms increased further during the second lockdown in Italy [16]. During the first lockdown, the same autistic adults experienced greater comfort and less tiredness [15], but during the second lockdown decreased comfort and gradually increased tiredness again [16]. When comparing two samples of autistic adults, one assessed before and the other during the pandemic, Martinez-Gonzales and colleagues [17] observed increased repetitive behaviours, more pronounced autism-related dysfunctions, including higher levels of aggression, irritability, and anxiety, as well as lower attention and higher hyperactivity in individuals assessed during the pandemic.

**Fig. 1:**
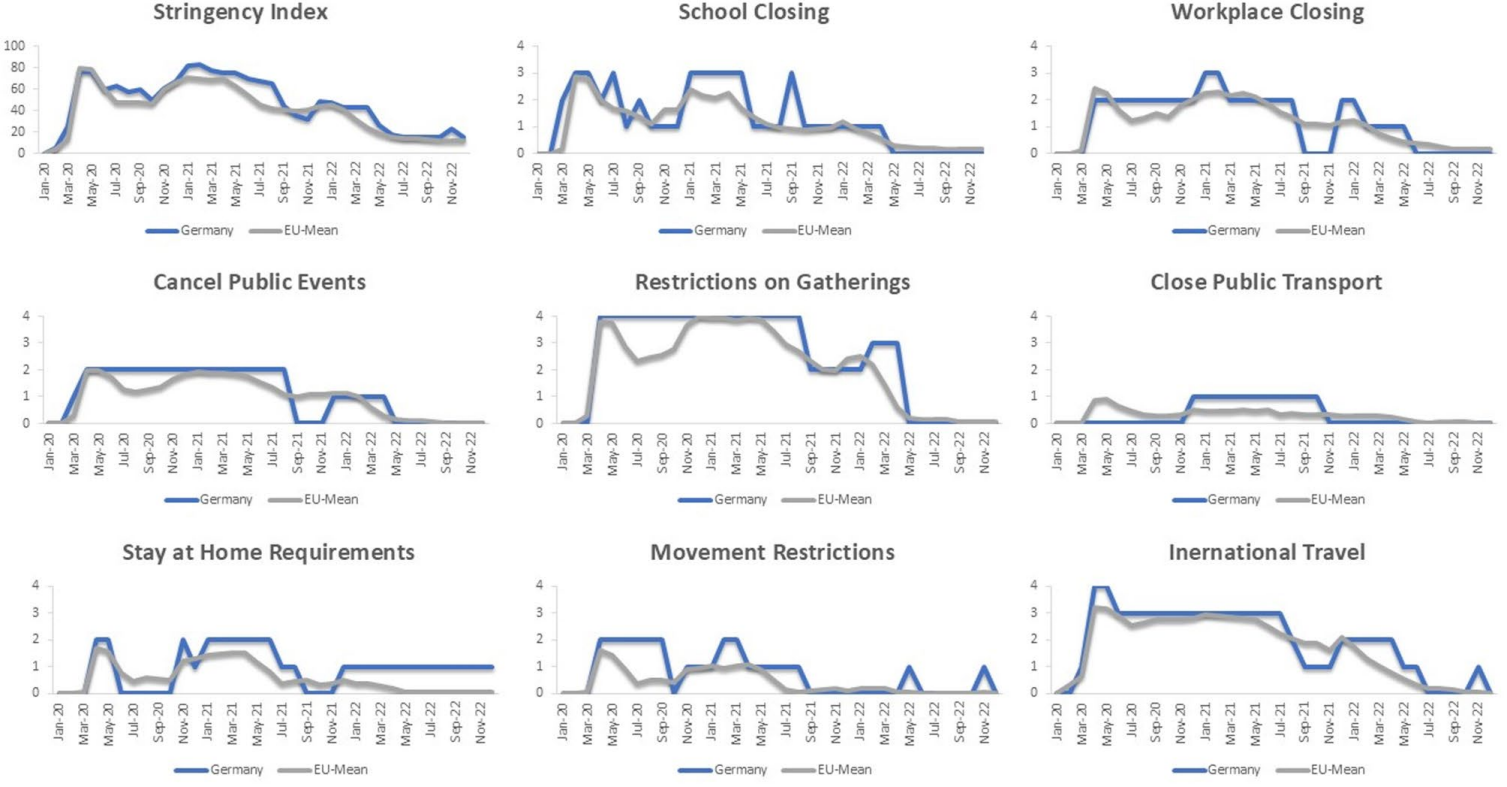
Overall stringency index and the eight indicators for containment and closure policies during the COVID-19 pandemic. Note. the overall stringency index is an aggregate of the eight indicators for containment and closure policies and an additional record of public information campaigns. The depicted EU-Mean includes the 27 Members of the European Union as well as the United Kingdom and Switzerland. The raw data was obtained from the Oxford Coronavirus Government Response Tracker [23]

Relatively few studies have examined predictors of psychopathological symptoms in autistic adults during the COVID-19 pandemic to this date and none of them were undertaken in Germany. A large longitudinal self-report study of 275 middle aged autistic adults in the United States investigating distress in relation to the COVID-19 pandemic found that higher pandemic-related distress was associated with increased symptoms of anxiety and depression. In addition, reporting COVID-19-related distress was more likely in autistic women and individuals with higher anxiety levels before onset of the pandemic [18]. Sex differences were also highlighted by Gaag and Wiingaarden-Cremers [19], who observed that autistic men reported less loneliness and greater use of online contacts and gaming, whereas autistic women experienced higher symptom-levels of anxiety, depression and suicidal behaviour. Another longitudinal study compared stress and loneliness in 448 autistic and 70 non-autistic adults in the Netherlands over the course of the pandemic. Autistic adults consistently reported higher levels of stress and loneliness compared to non-autistic adults at all time-points. A pre-pandemic mental health condition, low perceived social support and higher levels of worries in relation to COVID-19 were related to an increase in stress in autistic adults [20]. Taken together, most studies on autistic adults during the COVID-19 pandemic reported a greater deterioration of psychopathological symptoms and heightened levels of worry compared to non-autistic adults [14–16, 20]. However, some studies also reported some positive effects of lockdown for autistic adults [13, 21]. Few studies investigating predictors of psychopathological symptoms found striking differences related to personal factors such as sex and age. Few studies investigated the situation across the time-course of the COVID-19 pandemic and none of the studies reviewed above specifically addressed the situation in Germany. This represents a significant research gap, as the pandemic situation displayed high regional variability in terms of incidence rates and government-imposed regulations to try and control the pandemic. Compared to the EU average, confirmed deaths per 100,000 in Germany were somewhat low [22], while the extent of social restrictions was high (see Fig. 1). Especially school closures and restrictions in gathering were continuously high and above the EU average. Therefore, findings from other regions may not be generalisable directly. In addition, previous studies focussed predominantly on anxiety and depression, while other co-occurring psychopathological symptoms were examined less. From these research gaps, the overall aim of the present study was to expand previous research on predictors of psychopathological symptoms in autistic adults (e.g. IoU and sensory sensitivities) during the COVID-19 pandemic to the region of Eastern Germany and a broader spectrum of psychopathological symptoms (e.g. anxiety, depression, obsession-compulsion, and interpersonal sensitivity). More specifically, the first aim of this study was to describe and compare the impact of pandemic-related restrictions on daily life as well as global and specific psychopathological symptom severity in autistic and non-autistic adults during the COVID-19 pandemic in Eastern Germany.

We hypothesised (H1a) that autistic adults were affected more severely by sensory exposure through face coverings and interruptions of therapies, while non-autistic adults were affected more severely by changes in the social sector (H1b) due to reductions of social contacts and interruptions of activities with social contacts (e.g. working from home and online-classes, closure of kindergartens/childcare at home, cancellation of big events, closure of cultural institutions). In line with pre-pandemic findings, we expected higher global and specific psychopathological symptom severity, sensory sensitivity and IoU in autistic than non-autistic adults during the pandemic (H1c). In addition, we expected autistic adults to report a more pronounced negative change in physical and mental health compared to non-autistic adults (H1d). The second aim was to examine bivariate relationships among global psychopathological symptom severity, current infection risk, pandemic duration, pandemic-related concerns and restrictions, changes in overall physical and mental health, sensory sensitivity and IoU within each group and potential between-group differences within these bivariate relationships. We hypothesised (H2a) that global psychopathological symptom severity and being affected by the pandemic situation were related to current infection risk, pandemic duration, pandemic-related concerns and restrictions, changes in overall physical and mental health, sensory sensitivity and IoU in both groups. In addition, we hypothesised (H2b) that global psychopathological symptom severity was more strongly related to changes in overall physical health in autistic compared to non-autistic adults [24]. The third aim was to investigate predictors of psychopathological symptom severity in anxiety, depression, obsession-compulsion, and interpersonal sensitivity, all commonly co-occurring in autism, in autistic compared to non-autistic adults. Drawing on literature on symptoms of anxiety and depression in autism, predominantly focussed on in previous pre-pandemic autism-studies, we identified IoU and sensory sensitivities [4, 6–10] as potential predictors. Based on published pandemic-related research, additional predictors included overall change in mental health [5, 16], age [25, 26], sex [18, 19] and feeling affected by the pandemic (e.g [18]. investigating distress). To provide a more comprehensive perspective, the following variables were also considered as potential predictors: (1) changes in overall physical health, given that autistic adults have higher rates of co-occurring medical conditions [27] and, therefore, (2) concerns regarding COVID-19 infection. Furthermore, variations in incidence rates and COVID-19 stringency index were included as potential predictors due to their significant fluctuations during the survey period. In contrast to the second aim of the study (bivariate relationships), here we were interested in estimating the unique contribution of each predictor while controlling for the effects of all other predictors mentioned. We hypothesised for the group of autistic adults (H3a) to replicate IoU as unique predictor for symptoms of anxiety and depression, to replicate sensory sensitivities as unique predictor for symptoms of anxiety and extend this predictor to symptoms of depression, and to extend the relevance of both predictors to global psychopathological symptom severity, symptoms of obsession-compulsion, interpersonal sensitivity and global psychopathological symptom severity. Changes in overall physical health and concerns about COVID-19 infection were hypothesised (H3b) to constitute additional unique predictors of global and specific psychopathological symptoms in autistic adults. We further hypothesised (H3c) IoU and sensory sensitivities as unique predictors of anxious symptoms in non-autistic adults [12]. In a rather exploratory approach and drawing on previous evidence, we investigated IoU and sensory sensitivity as relevant unique predictors of global psychopathological symptom severity, symptoms of depression, obsession-compulsion and interpersonal sensitivity in non-autistic adults, and whether the proportion of explained variance differed between autistic and non-autistic adults. Finally, we hypothesised (H3d) overall change in mental health, age, sex, feeling affected by the pandemic, incidence rates and COVID-19 stringency index are additional predictors of global and specific psychopathological symptom severity in both groups.

## Methods

Data for the present study was collected as part of a broader research project aimed at improving anxiety diagnostics for autistic individuals. Several associated papers have already been published [5, 28, 29]. Individuals took part during the COVID-19 pandemic between May 2020 and May 2021 and completed questionnaires either online, via LimeSurvey [30], or using pen and paper. Individuals were compensated for their time with €10. Informed consent was obtained from each participant, and all study procedures adhered to the declaration of Helsinki. Ethical approval was granted by the ethics committee of TUD (approval code: EK 356092018).

### Participants

A sample of 173 individuals was studied, comprising 86 autistic adults aged 18–67 years (21 female, *M*_age_ = 33.49 years, *SD*_age_ = 13.32) and 87 non-autistic adults aged 18 to 70 years (21 female, *M*_age_ = 34.37 years, *SD*_*a*ge_ = 14.18). Autistic adults were patients of the Autism Clinic of the University Hospital Carl Gustav Carus of Technische Universität Dresden, Germany with a clinical diagnosis of childhood autism (F84.0) or Asperger’s syndrome (F84.5) according to the International Classification of Diseases 10 [31]. They were recruited via telephone, email, mail or through their therapists. Only individuals with an intelligence quotient (IQ) of 75 or higher were included in order to ensure comprehension of all relevant self-report questionnaires. For detailed information about the formal diagnostic procedures see Riedelbauch and colleagues [5]. Descriptive data on Intelligence Quotient (IQ) and Autism Diagnostic Observation Schedule 2 (ADOS-2) subscales of autistic adults are provided in Table S1. Non-autistic adults were recruited through various methods, including re-contacting individuals who participated in previous studies, sending invitation letters to randomly selected residents of Dresden (selected by the residents’ registration office), contacting staff of the University Hospital and privately advertising the study.

The groups of autistic and non-autistic individuals were matched in terms of sex, age and educational level (see Table 1). AQ scores were higher in the group of autistic adults (see Table 1). During the period with the highest incidence rates (110 to 197) in the present study, between November 2020 and February 2021, about 50% of the group of autistic adults completed the questionnaires, while less than 20% of the group of non-autistic adults did so. This resulted in higher incidence rates for the group of autistic adults compared to the group of non-autistic adults. However, no differences were observed between the groups in the Stringency Index, as social restrictions were not immediately linked to the incidence rates and were maintained even after incidence rates declined.

### Autism spectrum quotient (AQ)

Autism-like traits were quantified by the widely used Autism Spectrum Quotient [32]. The 50 items of this self-report questionnaire are rated on a 4-point Likert scale with scorings of 0 to 1, yielding a total score between 0 and 50, with a higher score indicating more autism-like traits. Internal consistency of the questionnaire for the current sample was acceptable (Cronbach’s alpha: autistic individuals = 0.72, non-autistic individuals = 0.68).

### Incidence rate

The 7-day incidence rate of new COVID-19 infections in Germany, monitored by the Robert Koch Institute, served as an objective measure of infection risk for the time of questionnaire completion by each individual.

### COVID-19 stringency index

The extent of social restrictions was quantified by the national stringency index as calculated by the Oxford Coronavirus Government Response Tracker (OxCGRT) project [33]. This index was calculated from the following nine metrics: school closures, workplace closures, cancellation of public events, restrictions on public gatherings, closures of public transport, stay-at-home requirements, public information campaigns, restrictions on internal movements and international travel controls. Each of the nine metrics ranges between 0 and 100, with higher scores indicating more stringency. The total stringency index was calculated as the mean score of the nine metrics.

### Impact of pandemic-related restrictions on daily life

An 8-item self-report questionnaire developed by the research team was used to assess the impact of pandemic-related restrictions on daily life. For 4 of the items, individuals rated (1) their concerns about infection, (2) the degree to which the pandemic situation affected them (not at all, slightly, moderately, strongly, very strongly) and (3) changes of their overall physical and (4) mental health (very worsened, worsened, not changed, improved, strongly improved) on 5-point Likert scales. For the other 4 items, individuals indicated (5) whether they or a family member was infected with COVID-19 (yes/no), (6) in which way they were most affected by the pandemic situation with multiple selections possible (*Loss of daily structure due to*: cancellation of big events, closure of cultural institutions, interruption of education/job, restrictions on freedom of travel, restrictions in leisure activities; *Changes in the social sector due to*: reduction of social contacts, increase in stress and tension in the family; Sensory exposure through face covering; *Interruption of therapies*; *Loss of financial support*; *Closure of kindergartens/childcare at home*), (7) which changes they found most difficult (*Everyday life*: adaptation to the restrictions due to COVID-19, return to a new normal life after lockdown, both equally difficult; *Job*: switch to the work at home, switching back to on-site work, both equally difficult) and (8) which positive changes they experienced during the pandemic (Financial savings; Reduced stress due to appointment cancellation; Reduction of stressful social contacts; Reduced sensory exposure). The frequencies of the answer options were calculated for the analyses.

### Glasgow sensory questionnaire (GSQ)

Sensory sensitivity was assessed by the 42-item self-report GSQ [34]. Individuals indicated their sensory sensitivity by rating the frequency of situations and phenomena on a 5-point Likert scale (scores: 0–4) with total scores’ ranging from 0 to 168 and with a higher score indicating more sensory sensitivity. The original English version had excellent internal consistency (*r* =.94) and was translated to German by the research team with permission of the authors (Robertson & Simmons, University of Glasgow). The German version presented good internal consistency (*r* =.88) [35]. The current sample also demonstrated good to excellent internal consistency for both groups (Cronbach’s alpha: autistic adults = 0.93; non-autistic adults = 0.86).

### Intolerance of uncertainty (IoU) scale

The 18-item self-report IoU Scale [36] assessed individuals’ intolerance of uncertainty on a 5-point Likert scale (scores: 1–5). Individuals rated whether they show non-acceptance and negative affect in the presence of uncertainty. Total scores ranged from 18 to 90, with higher scores indicating more intolerance. In the current sample, the scale demonstrated excellent internal consistency in both groups (Cronbach’s alpha: autistic adults = 0.92; non-autistic adults = 0.93).

### Symptom-Checklist 90-R (SCL-90-R)

The global and specific psychopathological symptom severity over the past seven days was assessed by the 90-item self-report Symptom-Checklist 90-R [37, 38]. Individuals rated their experience of psychopathological symptoms of somatization, obsessive-compulsive disorder, interpersonal sensitivity, depression, anxiety, hostility, phobic anxiety, paranoid ideation, and psychoticism on a 5-point Likert scale (scores: 0–4). The global psychopathological symptom severity was measured by the calculated Global Severity Index (GSI) across all items. Internal consistency was excellent in both groups (Cronbach’s alpha: autistic adults = 0.96; non-autistic adults = 0.97), consistent with the findings for the German version reported by Franke [37].

### Data analysis

For all analyses, raw scores of the measures were used. Missing item values in the questionnaires AQ, GSQ and SCL-90-R were imputed by replacing them with the individual’s mean item score on the respective subscale, provided that no more than two items were missing from that subscale. For group comparisons, relating to our first study aim, chi-square tests (sex, being affected by the pandemic and positive changes during the pandemic), t-tests (AQ, incidence rate, stringency index, pandemic duration, IoU, GSQ, GSI of the Symptom-Checklist and all the subscales) and U-tests (educational level, concerns about infection, change in overall physical health, change in overall mental health, affected by the pandemic situation) were run. The following parameters were used to determine the effect sizes: Φ (0.1 to 0.3 small effect, 0.3 to 0.5 moderate effect, ≥ 0.5 strong effect) for chi-square tests, *d* (0.2 to 0.4 small effect, 0.5 to 0.7 moderate effect, 0.8 to 1.0 large effect) for t-tests and *r* (0.1 to 0.3 small effect, 0.3 to 0.5 moderate effect, ≥ 0.5 strong effect) for U-tests [39]. Regarding our second study aim, examining the relationship among psychopathological symptoms and their potential predictors, the variables were analysed by spearman’s rank correlations for both groups respectively. Fisher’s Z was calculated for Spearman coefficients to compare the correlation coefficients between the groups as Fisher’s Z is more robust regarding type one errors [40]. For all of the above analyses the false discovery rate (FDR [41]), was used to control for type one errors in multiple testing. Addressing our third study aim, regression analyses were conducted to determine whether sensory sensitivity (GSQ), IoU, age, sex, impact of pandemic-related restrictions (i.e., change in overall physical health, change in overall mental health, affected by the pandemic situation) as well as incidence rates, COVID-19 stringency index and concerns about COVID-19 infection predict the global psychopathological symptom severity (GSI of the SCL-90-R) and specific psychopathological symptoms (SCL-90-R subscales anxiety, depression, obsession-compulsion, interpersonal sensitivity) with the largest between-group effect sizes (*d* > 0.50). Separate regression analyse were run for each of the dependent variables: global psychopathological symptom severity, obsession-compulsion, interpersonal sensitivity, depression and anxiety. Predictors were entered into the model in a single block (non-hierarchical method). In order to compare the explained proportion of the total variance as well as the predictive patterns between autistic and non-autistic adults, separate regression analyses were carried out for both groups. The effect sizes of the predictors were determined by f (0.02 to 0.14 small effect, 0.15 to 0.34 moderate effect, ≥ 0.35 strong effect) [39]. The following assumption checks were run before the regression analysis: outliers (< 3.5 SD), multicollinearity (Variance Inflation Factor < 10), autocorrelation (critical values according to [42]), homoscedasticity and multivariate normality (visual inspection). All above mentioned analyses were performed using IBM SPSS Statistics 28.

## Results

### Between-group differences in the impact of pandemic-related restrictions on daily life, global and specific psychopathological symptom severity

**Fig. 2:**
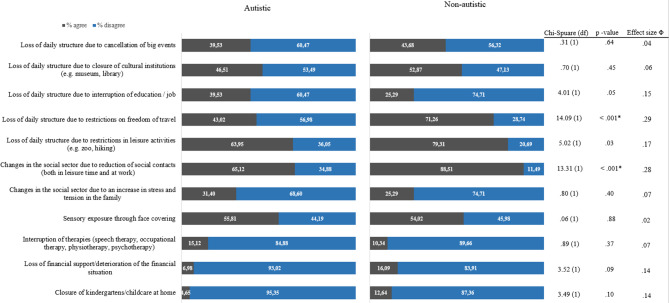
Frequencies and chi-square statistics about being affected by the pandemic

**Fig. 3:**
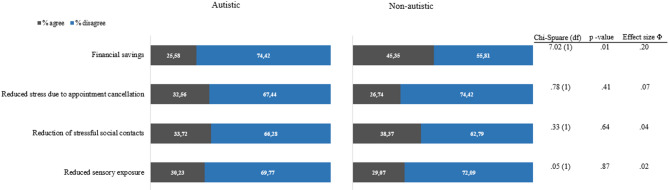
Frequencies and chi-square statistics about positive changes during the pandemic

Contrary to our hypothesis (H1a), autistic adults were not more affected by sensory exposure from face covering and interruption of therapies compared to non-autistic adults. Both groups reported being most affected by changes in the social domain due to the reduction of social contacts, and by the loss of daily structure as a result of the restriction of leisure activities. While the reduction in social contacts was identified as the most important for both groups, a difference in the agreement frequencies for this item was found, as hypothesised (H1b: autistic adults: 65.12% agreement; non-autistic adults: 88.51% agreement). An additional difference was found in the agreement frequencies regarding the loss of daily structure due to restriction of freedom of travel (autistic adults: 43.02% agreement; non-autistic dults:71.26% agreement). For both groups, the interruption of therapies, the loss of financial support and the closure of kindergartens/schools were the least relevant impacts. No between-group differences were observed concerning positive changes experienced during the pandemic. The agreement frequencies and the results of the chi-square tests are presented in Figs. 2 and 3.

As expected (H1c), autistic adults scored higher on the global psychopathological symptom severity and across all specific psychopathological symptom categories except for psychoticism symptoms. The most pronounced differences, as indicated by effect sizes (*d* > 0.5) were identified for the following specific psychopathological symptoms: obsession-compulsion, interpersonal sensitivity, depression and anxiety. However, after FDR correction, only the global psychopathological symptom severity remained significant. Less pronounced differences (*d* ≤ 0.5) were identified for the following specific psychopathological symptoms: somatization, hostility, phobic anxiety, paranoid ideation, psychoticism. Also in line with our hypothesis (H1c), autistic adults reported higher sensory sensitivity and IoU compared to non-autistic adults, with this difference remaining significant after FDR correction. Contrary to our hypothesis (H1d), individuals in both groups reported similar levels of change in overall physical and mental health. In addition, individuals in both groups reported similar levels of concerns about infections and how affected they were by the pandemic situation. Descriptive data and group comparison results are presented in Tables 1 and 2.

**Table 1:** Descriptive data and group comparisons

	Group of autistic adults	Group of non-autistic adults	Test Statistic	*p*-Value	Effect Size
*N*	86	87			
Sex, male, *n* (%)	65 (75.6)	66 (75.9)	*X*^2^ (1) = 0.002	*p* = 1.00	Φ = 0.003
Age, *M (SD)*	33.49 (13.32)	34.37 (14.18)	*t*(171) = − 0.42	*p* =.68	*d* = − 0.06
Education, *Md (SD)*	3 (1.14)	4 (0.95)	*Z* = −1.54	*p* =.13	*r* =.12
Autism-Spectrum Quotient^a^, *M (SD)*	32.82 (8.86)	17.49 (7.11)	*t*(171) = 12.56	*p* <.001*	*d* = 1.91
Incidence Rate, *M (SD)*	101.58 (58.84)	68.82 (58.75)	*t*(171) = 3.67	*p* <.001*	*d* = 0.56
COVID-19 Stringency Index, *M (SD)*	71.48 (11.40)	69.39 (11.69)	*t*(171) = 1.19	*p* =.24	*d* = 0.18
Pandemic duration in months since index case in Germany, *M (SD)*	9.76 (2.39)	9.60 (3.69)	*t*(147.62) = 0.34	*p* =.74	*d* = 0.05
Concerns about infection^a^, *Md (SD)*	8 (3.03)	9 (2.77)	*Z* = −1.84	*p* =.07	*r* =.14
Change in overall physical health^a^, *Md (SD)*	3 (0.63)	3 (0.65)	*Z* = − 0.96	*p* =.34	*r* =.07
Change in overall mental health^a^, *Md (SD)*	3 (0.79)	3 (0.63)	*Z* = −2.33	*p* =.02	*r* =.18
Affected by the pandemic situation^a^, *Md (SD)*	3 (1.19)	3 (1.10)	*Z* = − 0.37	*p* =.71	*r* =.03
Sensory Sensitivity ^a^, *M (SD)*	55.14 (24.34)	38.58 (14.92)	*t*(140.70) = 5.39	*p* <.001*	*d* = 0.82
IoU^a^, *M (SD)*	56.38 (14.95)	44.76 (13.99)	*t*(171) = 5.28	*p* <.001*	*d* = 0.80

*IoU* Intolerance of Uncertainty, *a* Raw score

*significant on the 0.05 level after FDR correction

**Table 2:** Group comparisons on the subscales of the SCL-90-R

	Group of a utistic adults	Group of non-autistic adults	Test Statistic	*p*-Value	Effect Size
*N*	83	84			
SCL-90 GSI^a^	59.70 (9.10)	52.60 (10.96)	*t*(160.27) = 4.56	*p* <.001*	*d* = 0.71
SCL-90, Somatization^b^	6.28 (6.28)	3.73 (3.70)	*t*(132.384) = 3.19	*p* =.002	*d* = 0.50
SCL-90, Obsessive-compulsive^b^	11.00 (6.75)	6.57 (6.08)	*t*(165) = 4.45	*p* =.001	*d* = 0.69
SCL-90, Interpersonal Sensitivity ^b^	7.59 (6.01)	4.48 (5.36)	*t*(165) = 3.54	*p* =.001	*d* = 0.55
SCL-90, Depression ^b^	12.35 (8.82)	7.83 (8.17)	*t*(165) = 3.43	*p* =.001	*d* = 0.53
SCL-90, Anxiety ^b^	6.67 (7.51)	3.38 (4.31)	*t*(130.42) = 3.47	*p* =.001	*d* = 0.54
SCL-90, Hostility ^b^	3.88 (3.94)	2.14 (2.98)	*t*(152.82) = 3.21	*p* =.002	*d* = 0.50
SCL-90, Phobic Anxiety ^b^	3.84 (4.07)	2.10 (3.67)	*t*(162.82) = 2.92	*p* =.004	*d* = 0.45
SCL-90, Paranoid Ideation ^b^	4.60 (4.30)	2.67 (3.53)	*t*(158.30) = 3.18	*p* =.002	*d* = 0.49
SCL-90, Psychoticism ^b^	4.60 (4.45)	3.10 (4.30)	*t*(165) = 2.23	*p* =.027	*d* = 0.35

*GSI* Global Severity Index

*significant on the 0.05 level after FDR correction

^a^T-Value

^b^Sum score

### Relationship among impact of pandemic-related restrictions, psychopathological symptoms and their predictors

In the group of non-autistic adults, being affected by the pandemic situation was positively correlated with the incidence rate, pandemic duration, concerns about infection and stringency index, as hypothesised (H2a). This indicates that non-autistic adults reported a greater impact of the pandemic when the incidence rate and social restrictions (stringency index) were higher, the pandemic lasted longer, and concerns about infections increased. In contrast and against our hypothesis (H2a), no correlations were observed in the group of autistic adults among being affected by the pandemic and these variables (incidence rate, pandemic duration, concerns about infection and stringency index). This unexpected finding suggests that autistic adults’ perception of being affected by the pandemic was less influenced by external pandemic-related metrics compared to the group of non-autistic adults. Even contrary to our hypothesis (H2a), no correlation was found between being affected by the pandemic with changes in overall physical and mental health, sensory sensitivity and IoU in either group. Another unexpected (H2a) pattern emerged regarding global psychopathological symptom severity (lower scores indicating less global psychopathological symptom severity), where negative correlations with both the stringency index and pandemic duration were observed in the group of autistic adults, though not significant after FDR correction. This trend suggests that longer pandemic duration and more social restrictions may have been associated with reduced global psychopathological symptom severity in autistic adults, possibly reflecting a relief from social and sensory demands during lock-down periods. For the group of non-autistic adults, global psychopathological symptom severity was not related to either the stringency index or the pandemic duration, another unexpected finding (H2a). Also contrary to our hypothesis (H2a), global psychopathological symptom severity was not related to incidence rate and concerns about infection, changes in overall physical and mental health, in both groups. As expected (H2a), global psychopathological symptom severity was related to sensory sensitivity and IoU in either group.

**Fig. 4:**
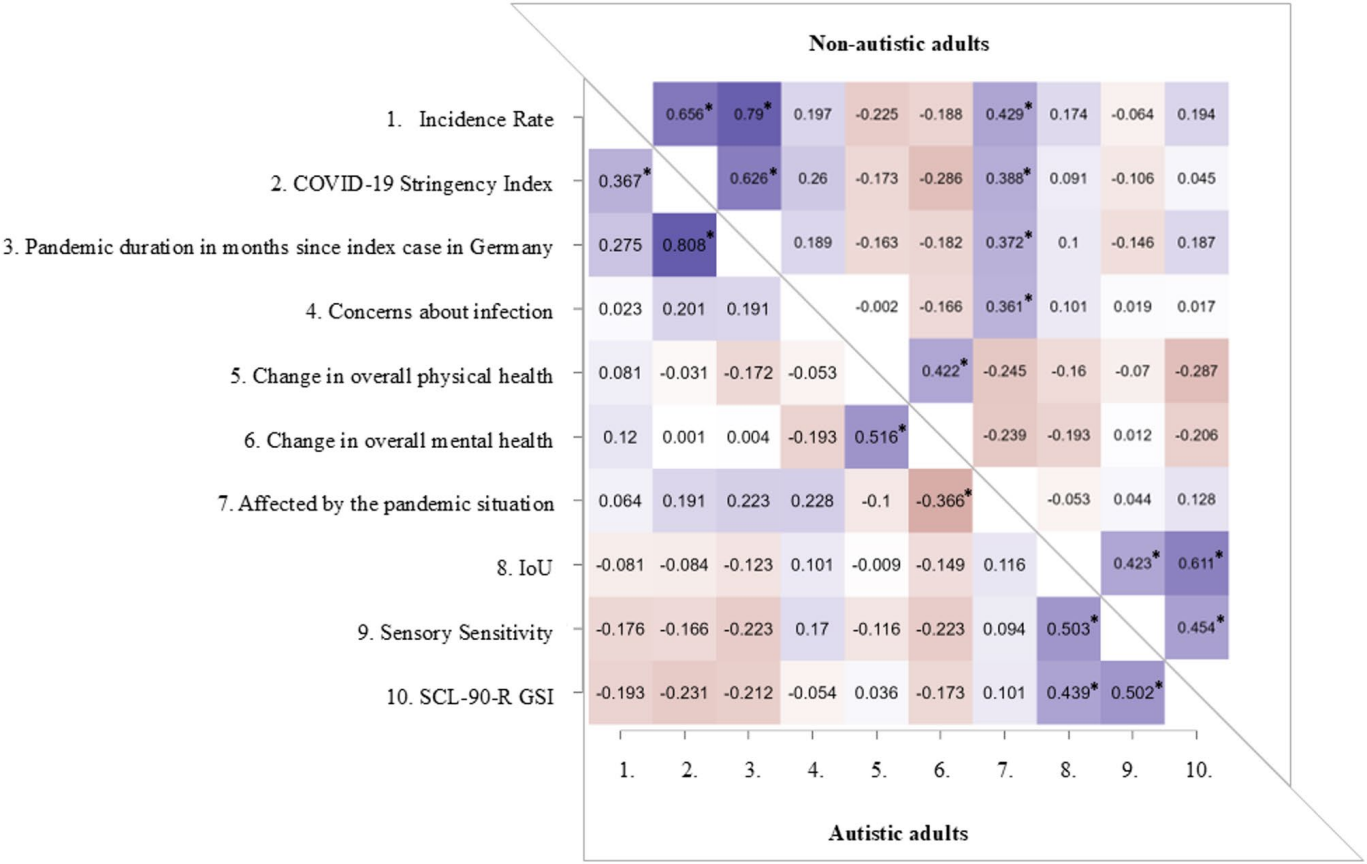
Heatmap with Spearman’s rank correlations for the groups of autistic and non-autistic adults. * significant on the .05 level after FDR correction

Even though the results of the Spearman rank correlation analysis revealed between-group differences in the relationships among pandemic-related variables and psychological outcomes, the hypothesised (H2b) stronger correlation between the psychopathological symptom severity and changes in overall physical health in autistic compared to non-autistic adults could not be confirmed by the present data. The only significant difference in correlation coefficients between groups was found for the relationship between the incidence rate and pandemic duration, which was stronger in the group of non-autistic adults. This discrepancy may be explained by the timing of data collection: from March to May 2021, during a rise in COVID-19 incidence, 25% of non-autistic adults completed the questionnaires compared to only 5% of autistic individuals. As expected, the global psychopathological symptom severity was positively correlated with the sensory sensitivity and IoU in both groups. The correlation coefficients are presented in Fig. 4and Table S2.

### Prediction of global and specific psychopathological symptom severity

Regression analyses were conducted using the enter method, with either global psychopathological symptom severity or one of the relevant subscales of psychopathological symptoms (specific psychopathological symptoms) as dependent variable. Predictors included sex, age, incidence rate, stringency index, concerns about infection, changes in overall physical health, changes in overall mental health, feeling affected by the pandemic situation, sensory sensitivity, and IoU. The results of the regression analyses for the global psychopathological symptom severity are presented in Table 3. The results of the obsession-compulsion scale, interpersonal sensitivity scale, depression scale and anxiety scale are presented in Table S3. The analyses revealed that the regression models explained a greater proportion of variance in the group of non-autistic compared to the group of autistic adults for the global and specific psychopathological symptom severity (obsession-compulsion, interpersonal sensitivity, depression and anxiety).

**Table 3:** Regression results for prediction of the global psychopathological symptom severity (Global severity index, GSI)

	Group of autistic adults					Group of non-autistic adults				
	B	SE	t	p	*f*^*2*^	B	SE	T	p	*f*^*2*^
*Global Severity Index*										
Sex	−3.58	2.00	−1.81	0.07	0.05	−6.12	2.18	−2.81	< 0.01**	0.11
Age	− 0.03	0.07	− 0.48	0.64	0.00	− 0.03	0.07	− 0.44	0.66	0.00
Incidence Rate	− 0.02	0.02	−1.47	0.15	0.03	0.02	0.02	0.69	0.50	0.01
COVID-19 Stringency Index	− 0.05	0.09	− 0.51	0.61	0.00	− 0.12	0.12	−1.05	0.30	0.02
Concerns about infection	− 0.39	0.28	−1.36	0.18	0.03	− 0.21	0.36	− 0.59	0.56	0.00
Change in overall physical health	3.43	1.55	2.21	0.03*	0.07	−1.49	1.54	− 0.96	0.34	0.01
Change in overall mental health	− 0.97	1.35	− 0.72	0.47	0.01	−1.53	1.62	− 0.94	0.35	0.01
Affected by the pandemic situation	1.03	0.75	1.37	0.18	0.03	1.24	1.02	1.22	0.23	0.02
Sensory Sensitivity	0.14	0.04	3.52	< 0.001**	0.17	0.23	0.07	3.34	< 0.01**	0.15
IoU	0.17	0.06	2.63	0.01**	0.10	0.38	0.08	5.03	< 0.001**	0.35

*p ≤.05, **p ≤.01

For the group of autistic adults, the model explained 38%, *F*(10,71) = 5.89, *p* <.001, of the total variance in the global psychopathological symptom severity, while for the group of non-autistic adults the model explained 49%, *F*(10,73) = 8.99, *p* <.001. Whilst only expected for autistic adults (H3a), sensory sensitivity (moderate effect for both groups) and IoU (small effect for the group of autistic adults, strong effect for the group of non-autistic adults) emerged as predictors for global psychopathological symptom severity in both groups. As expected (H3b), change in overall physical health was a further predictor for the group of autistic adults (small effect), whereas concerns about COVID-19 infection was not a predictor, contrary to our hypothesis. In turn, sex was another predictor for the group of non-autistic adults (small effect), although it was hypothesised that it is a predictor in both groups. The remaining predictors (overall change in mental health, age, feeling affected by the pandemic, incidence rates and COVID-19 stringency index) were not significant in either group, which was unexpected (H3d).

For symptoms of anxiety, the model explained 32%, *F*(10,71) = 4.80, *p* <.001, of the total variance in the group of autistic adults and 44%, *F*(10,73) = 7.46, *p* <.001, in the group of non-autistic adults. Sensory sensitivity (small effect) and change in overall physical health (moderate effect) were predictors for the group of autistic adults, as expected (H3a&b). In turn, that IoU and concerns about COVID-19 infection were not significant predictors in the group of autistic adults was unexpected (H3a&b). For the group of non-autistic individuals, sensory sensitivity (moderate effect) and IoU (all small effects) were predictors, as expected (H3c). For the group of autistic adults, feeling affected by the pandemic situation was another predictor, although this was expected to be a predictor in either group (H3d). Overall change in mental health and stringency index were further predictors for the group of non-autistic adults, although it was hypothesised that they are predictors in both groups (H3d). The remaining predictors (age, sex, incidence rates and COVID-19 stringency index) were not significant in either group, which was unexpected (H3d).

For depressive symptoms, the model explained only 23%, *F*(10,71) = 3.46, of the total variance in the group of autistic adults and 45%, *F*(10,73) = 7.68, *p* <.001, in the group of non-autistic adults. For the group of autistic adults, IoU (small effect) and change in overall physical health (small effect) were predictors, as expected (H3a&b). In turn, that sensory sensitivity and concerns about COVID-19 infection was not a significant predictor in the group of autistic adults was unexpected (H3a&b). For the group of non-autistic adults, IoU (moderate effect) was a predictor, which was unexpected (H3c). Overall change in mental health was a further predictor for the group of non-autistic adults, although it was hypothesised to be a predictor in either group (H3d). The remaining predictors (age, sex, feeling affected by the pandemic, incidence rates and COVID-19 stringency index) were not significant in either group, which was unexpected (H3d).

For the obsession-compulsion subscale, the model explaining 30% of total variance in the group of autistic adults, *F*(10,71) = 4.42, *p* <.001, and 43% in the group of non-autistic adults, *F*(10,73) = 7.20, *p* <.001. Again, sensory sensitivity (small effect for the group of autistic adults, strong effect for the group of non-autistic adults) and IoU (small effect for the group of autistic adults, moderate effect for the group of non-autistic adults) were predictors in both groups – although only expected for autistic adults (H3a). The remaining predictors were not significant - although we expected that changes in overall physical health and concerns about COVID-19 infection to be another predictor for the group of autistic adults (H3b) and that overall change in mental health, age, sex, feeling affected by the pandemic, incidence rates and COVID-19 stringency index to be predictors in either group (H3d).

For interpersonal sensitivity, the model explained 31%, *F*(10,71) = 4.72, *p* <.001, of total variance in the group of autistic adults and 38%, *F*(10,73) = 6.17, *p* <.001, in the group of non-autistic adults. Sensory sensitivity (small effect) and change in overall physical health (moderate effect) were predictors for the group of autistic adults, as expected (H3a&b). That IoU and concerns about COVID-19 infection was not a significant predictor in the group of autistic adults was unexpected (H3a&b). It was also unexpected that IoU (moderate effect) was a predictor for the group of non-autistic adults. Overall change in mental health was another predictor for the group of non-autistic adults, although it was hypothesised to be a predictor in either group (H3d). The remaining predictors (age, sex, feeling affected by the pandemic, incidence rates and COVID-19 stringency index) were not significant in either group, which was unexpected (H3d).

Notably, the symptom scores of the SCL-90-R generally increased with worse values of almost all predictors (more IoU, more sensory sensitivity, higher incidence, more restrictions, longer pandemic duration, more affected by the pandemic situation). In contrast, the opposite was found for the physical health of autistic adults, where higher scores on change in overall physical health (indicating no change or improvement of physical health) were associated with increased symptom scores of the SCL-90-R.

## Discussion

The present study aimed to compare autistic and non-autistic adults in terms of the impact of pandemic-related restrictions on daily life as well as global and specific psychopathological symptom severity. In addition, it sought to identify predictors of global and specific psychopathological symptom severity within both groups during the COVID-19 pandemic. Considering significant regional differences in incidence rates and in regulations, the study focussed on a sample of adults in Eastern Germany, individually controlling for incidence rates and public restrictions (quantified by the stringency index) at the time of data collection. This approach extends prior research and constitutes a key strength of the current study. Another key strength lies in the well-matched, comprehensively characterized sample with a broad age range. Unlike many studies conducted during the pandemic, the present study extended its scope beyond anxiety and depression symptoms to include other areas of psychopathology, namely obsessive-compulsive symptoms and interpersonal sensitivity.

In line with pre-pandemic evidence, autistic adults reported higher levels of IoU [43] and sensory sensitivity [44, 45], as well as global psychopathological symptom severity [46] compared to non-autistic adults during the COVID-19 pandemic. Also in line with previous literature, higher levels of specific psychopathological symptoms were observed in autistic compared to non-autistic adults particularly in anxious, depressive and obsessive-compulsive symptoms and interpersonal sensitivity [2, 3]. Unexpectedly and diverging from prior findings, no between-group differences emerged in retrospective self-reports of changes in overall physical or mental health from before to during the COVID-19 pandemic. Previous studies have yielded mixed findings, with some reporting decreases [13] and others observing increases in psychopathology among autistic adults, often to a significantly greater extent than in non-autistic adults [14–16]. One potential explanation for these discrepancies is that autistic adults may have had their essential needs met during the pandemic and, therefore, reported no more changes in overall physical or mental health than the non-autistic adults. For instance, disruptions to therapeutic interventions did not appear to affect autistic adults more than non-autistic ones. It is also noteworthy that this study sample exhibited a mean IQ within the average range. A sample containing individuals with an IQ below 75 might have shown more struggles to pandemic-related changes. Retrospective self-assessments, a limitation of this study, may have further influenced these results.

With regard to experiences during the COVID-19 pandemic in both groups, reductions in social contacts and restrictions on freedom of travel emerged as most significant challenges for both groups. Notably, non-autistic adults reported being at least as impacted as autistic adults. Consistent with previous studies, autistic adults expressed concerns about losing daily structure during the COVID-19 pandemic [14]. Non-autistic adults, however, reported similar concerns. Unexpectedly, sensory sensitivities related to face coverings affected both groups to a similar extent, despite autistic adults reporting higher sensory sensitivity on the GSQ. Notably, the standard deviation of the GSQ responses in the group of autistic adults was twice as high as that in the group of non-autistic adults, suggesting that some autistic adults may have faced substantial challenges with sensory stimuli during the pandemic, while others experienced fewer difficulties. This latter observation aligns with prior research [14, 15]. Reduced loads could be attributed to environmental factors such as reduced noise due to fewer cars, planes and other means of transportation, as well as quieter outdoor spaces due to fewer people being outside and fewer obligations to leave home, such as commuting to work. It was also unexpected that, for autistic adults, the degree to which they felt affected by the pandemic was not associated with incidence rate, stringency index, pandemic duration, or concerns about infections. This finding suggests that autistic adults were less affected by health-related factors of the pandemic. In addition, the extent of social restrictions (stringency index) appears to be not related to changes in overall mental health experienced by autistic adults. On the contrary, higher extent of social restrictions (stringency index) and a longer pandemic duration seemed to be related to less global psychopathological symptom severity. However, this result does not remain significant after correction for multiple testing and was not a significant predictor for global psychopathological symptom severity in the regression model. A possible explanation for the trend noticed in the correlation could be that autistic adults experienced relief during lockdowns due to the reduction of social and sensory stimuli such as the ones named above. However, the result of the regression indicates that the stringency index is no strong predictor that explains additional variance above other factors.

The regression analyses revealed a similar picture for the global and specific psychopathological symptom severity. In the group of autistic adults, sensory sensitivities and IoU emerged as predictors in most regressions. These findings replicate and expand previous research on anxious and depressive symptoms [4–10] to the global psychopathological symptom severity, obsessive-compulsive symptoms and interpersonal sensitivity. A similar pattern was found in the group of non-autistic adults, where sensory sensitivities and IoU - despite being lower than in the group of autistic adults – emerged as significant predictors explaining a substantial proportion of the variance in psychopathological symptoms. This finding was not unexpected at all, as previous research suggested that IoU is a rather global predictor that is relevant for a broader range of individuals and psychopathologies [4–10, 12]. However, the effect size of IoU as predictor for non-autistic adults was unexpected high (large effect size). Speculatively, autistic and non-autistic adults may be affected by different aspects of uncertainty, which the questionnaire used in this study may not adequately distinguish. For example, the statement “Unforeseen events upset me greatly”, may have led to different associations for individuals in the two groups. Autistic adults may have associated the question about uncertainty more often with everyday unforeseen events (e.g., train delays or uncertainties in social interactions). These types of events decreased during the pandemic, which may have reduced their impact on psychopathological symptoms in autistic adults. In contrast, non-autistic adults may have associated uncertainty more with pandemic-related events (e.g., new restrictions due to rising infection rates), which increased during the pandemic and may have intensified their impact on psychopathological symptoms in non-autistic adults. However, this is just speculation and future studies should investigate in more detail how exactly the IoU construct differs between autistic and non-autistic adults and how this can be assessed. An explanation for the stronger predictive association of IoU for the severity of global psychopathological symptoms in non-autistic adults, despite higher overall IoU scores in autistic adults, may be related to the different predictive patterns of IoU and sensory sensitivity in the two groups. As demonstrated by Riedelbauch and colleagues [5] sensory sensitivity plays a stronger predictive role in the context of autism, which in turn diminishes the predictive power of IoU in autistic individuals, as IoU and sensory sensitivity are interrelated.

Another predictor identified for overall psychopathological symptom severity was changes in overall physical health. While this factor has been largely overlooked in prior research on the topic, the present findings underscore its relevance. Within the group of non-autistic adults, a decrease in overall physical health tended to increase global psychopathological symptoms, a relation that remained not significant after FDR correction in the correlation. Moreover, overall physical health was not a significant predictor of global psychopathological symptom severity in the regression model for non-autistic adults. However, in the group of autistic adults, the regression model revealed an unexpected pattern: higher scores - with medium scores reflecting stagnation and high scores reflecting improvement in physical health - were associated with an increase in psychopathological symptoms. To our knowledge, no study has identified such a pattern to this date. Speculatively, this could be explained by the possibility that autistic adults may have rated their psychopathological symptom severity as lower with decreasing physical health, as physical complaints may have become more salient. However, it could also be explained by the other way round, as autistic adults may have rated their physical health as improved with increasing psychopathological symptom severity, as psychopathological symptoms may have become more salient. However, this does not explain why this pattern was observed exclusively in autistic adults and not in non-autistic adults. Future studies should aim to replicate this relationship and examine potential explanatory models.

Sex did not emerge as a predictor for any outcomes in the group of autistic adults, contrasting with prior studies [18, 19]. The small subgroup of women included in the autistic group may have limited the ability to detect sex-specific differences. Future research interested in sex differences should aim to include larger samples with a more balanced gender distribution to further explore these differences.

Similarly, previous results on the effects of age on psychopathological symptoms could not be replicated in this study. A large part of our sample was surveyed in the autumn and Christmas period of 2020, a time window in which Best and colleagues [47] found decreased age differences in symptoms of anxiety and depression as compared to the beginning of the pandemic. That could be an explanation for the lack of age effects in the present study.

### Limitations

The main limitation of the present study is a missing measurement invariance of the SCL-90-R subscales between the groups. To determine whether potential methodological explanations for between-group differences in psychopathological symptoms existed in addition to substantive ones, we tested the measurement invariance (i.e. psychometric equivalence in the construct measured by the questionnaire in both groups) for the SCL-90-R subscales. Our results revealed no configurational, metric or scalar invariance of the SCL-90-R subscales between the groups. Detailed information on the analyses of measurement invariance is provided in the online supplementary material. Consequently, observed group differences in predictors may be attributable not only to substantive differences but also to psychometric differences in the underlying construct. However, the measurement invariance should be tested again in an appropriately large sample of autistic and non-autistic adults, to enable more reliable interpretations.

A further methodological limitation of the present study is the cross-sectional nature of the study which does not allow conclusions about causal links. Furthermore, our results may have been influenced by recall bias in the retrospective self-assessments, as negative emotional valence enhances recapitulation [48].

In addition to the methodological limitations of the present study, the characteristics of the sample impose further limitations. While the gender imbalance in the study sample may have hindered the detection of sex-specific differences, the exclusion of individuals with an IQ below 75 could have obscured difficulties in coping with pandemic-related changes.

## Conclusion

In summary, the group of autistic adults appeared less affected during the COVID-19 pandemic than expected, with notable differences in the impact of pandemic-related challenges between groups. Predictors for psychopathological symptoms differed in parts between groups, especially with regard to changes in overall physical health. Longitudinal studies comparing psychopathological symptoms during and after the COVID-19 pandemic are needed to provide valuable insights into long-term trajectories of global and specific psychopathological symptom severity.

## Supplementary Information

Supplementary Material 1.

## Data Availability

The data and materials are available from the authors upon reasonable request.

## Abbreviations

AQ: Autism Spectrum Quotient
CFI: Comparative Fit Index
FDR: False Discovery Rate
GSI: Global Severity Index
GSQ: Glasgow Sensory Questionnaire
IoU: Intolerance of Uncertainty
IQ: Intelligence Quotient
RMSEA: Root Mean Square Error of Approximation
SCL-90-R: Symptom-Checklist 90-R
SRMR: Standardized Root Mean Square Residual
