# Supplementary material for: The impact of the COVID-19 pandemic on the well-being of autistic and non-autistic adults in Eastern Germany
Source: BMC Psychiatry. 2025 Oct 2;25:915. doi: 10.1186/s12888-025-07430-x (PMC12490142; doi:10.1186/s12888-025-07430-x)
Supplement: Supplementary file 1 — Supplementary Material 1. [file 12888_2025_7430_MOESM1_ESM.pdf]

The impact of the COVID-19 pandemic on the well-being of autistic and non-autistic adults in Eastern Germany.

Supplementary material

Table S1. Descriptive data on Intelligence Quotient (IQ) and Autism Diagnostic Observation Schedule 2 (ADOS-2) subscales of autistic adults.

	n	Mean	Standard deviation
FIQ[a]	77	103.78	15.99
ADOS-2 COM[b]	81	4.12	1.83
ADOS-2 SA[c]	82	7.76	2.75
ADOS-2 CREA[d]	79	1.18	.68
ADOS-2 RRB[e]	82	1.06	1.09

[a]Full-Scale Intelligence Quotient assessed by HAWIK-IV, WIE, WAIS-IV, HAWIE-R, HAWIK-III and CFT1.

[b]ADOS-2 Communication subscale.

[c]ADOS-2 Reciprocal Social Interaction subscale.

[d]ADOS-2 Creativity subscale.

[e]ADOS-2 Restricted and Repetitive Behaviours subscale. Higher scores on ADOS-2 subscales indicate higher symptom level of the autistic spectrum.

The impact of the COVID-19 pandemic on the well-being of autistic and non-autistic adults in Eastern Germany.

Table S2. Spearman's rank correlations for the group of autistic and non-autistic adults with Fisher's Z for comparisons between the correlation coefficients.

Groups of autistic and non-autistic adults	1.	2.	3.	4.	5.	6.	7.	8.	9.	10.
1. Incidence Rate										
Autistic	-	.367*	.275	.023	.081	.120	.064	-.081	-.176	-.193
Non-autistic	-	.656*	.790*	.197	-.225	-.188	.429*	.174	-.064	.194
Difference between correlation coefficients, Z	-	-2.590	-5.099*	-1.132	2.002	2.010	-2.546	-1.659	-.735	-2.490
2. COVID-19 Stringency Index										
Autistic	-	-	.808*	.201	-.031	.001	.191	-.084	-.166	-.231
Non-autistic	-	-	.626*	.260	-.173	-.286	.388*	.091	-.106	.045
Difference between correlation coefficients, Z	-	-	2.497	-.404	.926	1.909	-1.394	-1.132	-.394	-1.781
3. Pandemic duration in months since index case in Germany										
Autistic	-	-	-	.191	-.172	.004	.223	-.123	-.223	-.212
Non-autistic	-	-	-	.189	-.163	-.182	.372*	.100	-.146	.187
Difference between correlation coefficients, Z	-	-	-	.008	-.060	1.217	-1.059	-1.448	-.517	-2.569
4. Concerns about infection										
Autistic	-	-	-	-	-.053	-.193	.228	.101	.170	-.054
Non-autistic	-	-	-	-	-.002	-.166	.361*	.101	.019	.017
Difference between correlation coefficients, Z	-	-	-	-	-.328	-.179	-.943	-.003	.989	-.447
5. Change in overall physical health										
Autistic	-	-	-	-	-	.516*	-.100	-.009	-.116	.036
Non-autistic	-	-	-	-	-	.422*	-.245	-.160	-.070	-.287
Difference between correlation coefficients, Z	-	-	-	-	-	.780	.968	.982	-.304	2.105

6. Change in overall mental health										
Autistic	-	-	-	-	-	-	-.366*	-.149	-.223	-.173
Non-autistic	-	-	-	-	-	-	-.239	-.193	.012	-.206
Difference between correlation coefficients, Z	-	-	-	-	-	-	-.905	.293	-1.541	.217
7. Affected by the pandemic situation										
Autistic	-	-	-	-	-	-	-	.116	.094	.101
Non-autistic	-	-	-	-	-	-	-	-.053	.044	.128
Difference between correlation coefficients, Z	-	-	-	-	-	-	-	1.096	.326	-.173
8. IoU										
Autistic	-	-	-	-	-	-	-	-	.503*	.439*
Non-autistic	-	-	-	-	-	-	-	-	.423*	.611*
Difference between correlation coefficients, Z	-	-	-	-	-	-	-	-	.658	-1.519
9. Sensory Sensitivity										
Autistic	-	-	-	-	-	-	-	-	-	.502*
Non-autistic	-	-	-	-	-	-	-	-	-	.454*
Difference between correlation coefficients, Z	-	-	-	-	-	-	-	-	-	.395
10. SCL-90-R GSI										

* significant on the .05 level after FDR correction

Table S3. Regression results for prediction of the Obsession-Compulsion Scale, Interpersonal Sensitivity Scale, Depression Scale and Anxiety Scale of the SCL-90-R.

	Group of autistic adults					Group of non-autistic adults				
	B	*SE*	*t*	*p*	f^2	*B*	*SE*	*t*	*p*	f^2
Anxiety										
Sex	.35	1.71	.20	.84	.00	-.70	.90	-.77	.44	.01
Age	-.07	.06	-1.27	.21	.02	-.02	.03	-.75	.46	.01
Incidence Rate	-.01	.01	-.99	.33	.02	.01	.01	1.20	.24	.02
COVID-19 Stringency Index	-.12	.08	-1.54	.13	.03	-.10	.05	-2.13	.04*	.06
Concerns about infection	-.20	.24	-.80	.42	.04	.18	.15	1.23	.22	.02
Change in physical health	5.10	1.34	3.81	<.001**	.21	-.38	.64	-.60	.55	.00
Change in mental health	.28	1.17	.24	.81	.00	-1.85	.67	-2.76	<.01**	.10
Affected by the pandemic situation	1.53	.65	2.35	.02*	.08	.17	.42	.40	.69	.00
Sensory Sensitivity	.07	.03	2.14	.04*	.07	.11	.03	3.72	<.001**	.19
IoU	.07	.06	1.30	.20	.03	.09	.03	3.00	<.01**	.12
Depression										
Sex	2.33	2.13	1.10	.28	.02	-2.27	1.70	-1.34	.19	.02
Age	-.05	.07	-.76	.45	.01	-.05	.05	-.92	.36	.01
Incidence Rate	-.02	.02	-.91	.37	.01	.01	.02	.64	.53	.01
COVID-19 Stringency Index	-.03	.10	-.32	.75	.00	-.14	.09	-1.56	.12	.03
Concerns about infection	-.44	.30	-1.43	.16	.03	-.07	.28	-.26	.80	.00
Change in physical health	4.87	1.67	2.92	<.01**	.12	-1.85	1.20	-1.55	.13	.03
Change in mental health	-1.61	1.45	-1.11	.27	.02	-2.51	1.26	-1.99	.05*	.06
Affected by the pandemic situation	1.36	.81	1.69	.10	.04	.34	.79	.43	.67	.00
Sensory Sensitivity	.06	.04	1.36	.18	.03	.10	.05	1.94	.06	.05
IoU	.15	.07	2.16	.03*	.07	.29	.06	4.91	<.001**	.33

The impact of the COVID-19 pandemic on the well-being of autistic and non-autistic adults in Eastern Germany.

	Group of autistic adults					Group of non-autistic adults				
	B	*SE*	*t*	*p*	f^2	*B*	*SE*	*t*	*p*	f^2
Obsessive-Compulsive										
Sex	.48	1.56	.31	.76	.00	-2.32	1.29	-1.81	.08	.04
Age	-.05	.05	-.93	.36	.01	-.06	.04	-1.60	.12	.03
Incidence Rate	-.01	.01	-.36	.72	.00	.01	.01	.86	.39	.01
COVID-19 Stringency Index	-.00	.07	-.03	.97	.00	-.09	.07	-1.32	.19	.02
Concerns about infection	-.07	.22	-.32	.75	.00	-.06	.21	-.28	.78	.00
Change in physical health	2.47	1.22	2.02	.05	.06	-.72	.91	-.80	.43	.01
Change in mental health	-.32	1.06	-.30	.76	.00	-1.47	.95	-1.54	.13	.03
Affected by the pandemic situation	.56	.59	.95	.35	.01	-.43	.60	-.72	.48	.01
Sensory Sensitivity	.08	.03	2.68	<.01**	.10	.17	.04	4.07	<.001**	.23
IoU	.16	.05	3.10	<.01**	.14	.15	.05	3.34	<.01**	.15
Interpersonal Sensitivity										
Sex	-.01	1.37	-.01	.99	.00	-.71	1.17	-.60	.55	.01
Age	.02	.05	.46	.65	.00	-.01	.04	-.27	.79	.00
Incidence Rate	-.01	.01	-1.10	.27	.02	.01	.01	.48	.63	.00
COVID-19 Stringency Index	-.00	.06	-.03	.98	.00	-.08	.06	-1.30	.20	.02
Concerns about infection	-.37	.20	-1.89	.06	.05	-.08	.19	-.40	.69	.00
Change in physical health	3.66	1.07	3.42	<.01**	.16	-.13	.83	-.15	.88	.00
Change in mental health	-1.22	.93	-1.31	.20	.02	-2.32	.87	-2.66	.01**	.10
Affected by the pandemic situation	.69	.52	1.32	.19	.02	-.06	.55	-.12	.91	.00
Sensory Sensitivity	.09	.03	3.13	<.01**	.14	.07	.04	1.82	.07	.04
IoU	.06	.04	1.35	.18	.02	.18	.04	4.39	<.001**	.26

$^*p \leq .05$, $^{**}p \leq .01$

SCL-90-R Measurement Invariance

Although the sample was rather small to test measurement invariance, this was important since no study to this date examined the factor structure of the SCL-90-R in autistic adults despite its frequent use in autism research [1–4]. First, Confirmatory Factor Analysis (CFAs) were conducted separately for each group. Due to the small sample size, separate CFAs were performed only for the relevant subscales of the Symptom-Checklist, each with one latent factor. Next, metric and scalar measurement invariance were examined for the entire sample. Model fits were evaluated according to chi-square exact-fit test, comparative fit index (CFI) ≥ 0.90 [5], root mean square error of approximation (RMSEA) ≤ 0.08 [6] and standardized root mean square residual (SRMR) ≤ 0.08 [7].The analysis of measurement invariance was conducted using the lavaan package in RStudio (2023.06.0). Consistent with previous findings that have already shown that the factor structure of the SCL-90 could not be confirmed [8], particularly in samples of individuals with psychiatric disorders [9–11], our results could not confirm the factor structure within each group and revealed no configurational, metric or scalar invariance of the SCL-90-R subscales between the groups (see Table S4) and, therefore, indicate, that there might be methodological explanations for differences in psychopathological symptoms in addition to substantive ones.

Table S4. Results of the measurement invariance tests.

	Chi^2	CFI	RMSEA	SRMR	Model comp	Δ Chi^2	Decision
Anxiety							
Autistic – CFA	81.19**	.90	.13**	.06	-	-	Reject
Non-autistic – CFA	103.89**	.77	.15**	.09	-	-	Reject
M1: Configurational invariance	185.08**	.85	.14	.07	-	-	Reject
M2: Metric invariance	200.86**	.84	.14	.09	M1	15.78	Reject
M3: Scalar invariance	206.20**	.85	.13**	.10	M2	5.34	Reject
Depression							
Autistic – CFA	137.12**	.80	.12**	.08	-	-	Reject
Non-autistic – CFA	223.45**	.75	.17**	.09	-	-	Reject
M1: Configurational invariance	360.57**	.77	.15	.08	-	-	Reject
M2: Metric invariance	386.49**	.76	.14	.11	M1	25.92*	Reject
M3: Scalar invariance	414.17**	.74	.14**	.11	M2	27.68*	Reject
Obsession-Compulsion							
Autistic – CFA	46.32	.94	.06	.07	-	-	Accept
Non-autistic – CFA	173.17**	.64	.22**	.12	-	-	Reject
M1: Configurational invariance	219.49**	.74	.16**	.09	-	-	Reject
M2: Metric invariance	235.74**	.72	.15**	.11	M1	16.24	Reject
M3: Scalar invariance	270.59**	.68	.16**	.12	M2	34.86**	Reject
Interpersonal Sensitivity							
Autistic – CFA	28.14*	.90	.11	.07	-	-	Reject
Non-autistic – CFA	22.65	.96	.09	.06	-	-	Accept
M1: Configurational invariance	50.79**	.94	.10*	.06	-	-	Reject
M2: Metric invariance	62.29**	.92	.10*	.09	M1	11.50	Reject
M3: Scalar invariance	75.97**	.90	.10*	.09	M2	13.67*	Reject

*p ≤ .05, **p ≤ .01

References

[1] Bruggink A, Huisman S, Vuijk R, Kraaij V, Garnefski N. Cognitive emotion regulation, anxiety and depression in adults with autism spectrum disorder. Research in Autism Spectrum Disorders 2016;22:34–44. https://doi.org/10.1016/j.rasd.2015.11.003.

[2] Lever AG, Geurts HM. Psychiatric Co-occurring Symptoms and Disorders in Young, Middle-Aged, and Older Adults with Autism Spectrum Disorder. J Autism Dev Disord 2016;46:1916–30. https://doi.org/10.1007/s10803-016-2722-8.

[3] Morrier MJ, Ousley OY, Caceres-Gamundi GA, Segall MJ, Cubells JF, Young LJ, et al. Brief Report: Relationship Between ADOS-2, Module 4 Calibrated Severity Scores (CSS) and Social and Non-Social Standardized Assessment Measures in Adult Males with Autism Spectrum Disorder (ASD). J Autism Dev Disord 2017;47:4018–24. https://doi.org/10.1007/s10803-017-3293-z.

[4] Torenvliet C, Groenman AP, Radhoe TA, Agelink van Rentergem JA, Geurts HM. One size does not fit all: An individualized approach to understand heterogeneous cognitive performance in autistic adults. Autism Research 2023;16:734–44. https://doi.org/10.1002/aur.2878.

[5] Byrne BM. Structural equation modeling with EQS and EQS/Windows: Basic concepts, applications, and programming. Sage; 1994.

[6] Brown MW, Cudeck R. Alternative ways of assess- ing model fit. Testing Structural Equation Models 1993;154:136–62.

[7] Hu L, Bentler PM. Cutoff criteria for fit indexes in covariance structure analysis: Conventional criteria versus new alternatives. Structural Equation Modeling: A Multidisciplinary Journal 1999;6:1–55. https://doi.org/10.1080/10705519909540118.

[8] Schmitz N, Hartkamp N, Kiuse J, Franke GH, Reister G, Tress W. The Symptom Check-List-90-R (SCL-90-R): A German validation study. Qual Life Res 2000;9:185–93. https://doi.org/10.1023/A:1008931926181.

[9] Bergly TH, Nordfjærn T, Hagen R. The dimensional structure of SCL-90-R in a sample of patients with substance use disorder. Journal of Substance Use 2014;19:257–61. https://doi.org/10.3109/14659891.2013.790494.

[10] Dinning WD, Evans RG. Discriminant and Convergent Validity of the SCL-90 in Psychiatric Inpatients. Journal of Personality Assessment 1977;41:304–10. https://doi.org/10.1207/s15327752jpa4103_13.

[11] Hardt J, Gerbershagen HU, Franke P. The symptom check-list, SCL-90-R: its use and characteristics in chronic pain patients. European Journal of Pain 2000;4:137–48. https://doi.org/10.1053/eujp.2000.0162.
